# Infectious complications after induction chemotherapy with FLAI(E) in newly diagnosed AML, omitting antibacterial prophylaxis

**DOI:** 10.1038/s41375-023-01930-z

**Published:** 2023-05-17

**Authors:** Adele Santoni, Margherita Malchiodi, Elisabetta Zappone, Anna Sicuranza, Monica Bocchia

**Affiliations:** University of Siena, Azienda Ospedaliera Universitaria Senese, Hematology Unit, Siena, Italy

**Keywords:** Acute myeloid leukaemia, Chemotherapy

## To the Editor:

We recently read the very interesting paper by Walti et al. [1] in which they investigated infections occurred in Acute Myeloid Leukemia (AML) patients after induction chemotherapy. Between 2006 and 2018 they retrospectively collected data of newly diagnosed (ND) AML treated with cladribine, high dose cytarabine, G-CSF and dose escalated mitoxantrone (CLAG-M) or standard dose cytarabine plus anthracycline (7 + 3). Authors confirmed that infections are very common after intensive induction chemotherapy, reporting a similar overall infection rate in both cohorts, albeit due to the increased incidence of fever of unknown origin (FUO) in the 7 + 3 cohort, and an increased incidence of microbiologically documented infections after CLAG-M, the latter probably due to the higher cytarabine dose and the deeper immunosuppression. They also confirmed that infections are the most frequent cause of morbidity in the setting of neutropenic AML patients undergoing intensive induction. We would like to report our retrospective results in 93 ND AML patients ≥18 years old (excluding promyelocytic leukemia) treated between 2015 and 2022 with a fludarabine-based induction resulting in neutropenia (neutrophiles count <500/mmc) ≥7 days. Chemotherapy was administered intravenously: idarubicine 6 mg/m^2^/day (days 1,3,5), fludarabine 25 mg/m^2^/day, high dose cytarabine 2000 mg/m^2^/day, ± etoposide 100 mg/m^2^/day (FLAI/FLAIE), on days 1–5. We describe infections occurring during induction from start of chemotherapy till up to the following 90 days with a median of 45 days of observation. In the majority of patients (82/93, 88.2%) censoring was the next treatment. Our population received standard antifungal prophylaxis (oral posaconazole 300 mg daily), without receiving antibacterial prophylaxis due to the high rates of local fluoroquinolone resistance. The baseline characteristics of our population and the infection events occurred are summarized in Table 1. In our analysis 84/93 (90.3%) patients presented fever, defined as a single temperature measurement of ≥38.5 °C or a temperature of ≥38.0 °C in two consecutive detections over a 1-hour period. Overall, we observed 45/93 (48.4%) microbiologically documented infections, with 34/93 (36.6%) bacterial infections, 10/93 (10.8%) fungal and 1/93 (1.1%) viral. Instead, in those 23/93 (24.7%) patients with a radiological documented infection the incidence of bacterial and fungal documentation was similar: 11/93 (11.8%) and 10/93 (10.8%), respectively.

**Table 1:** Baseline, treatment characteristics and infection events.

Baseline characteristics	Newly diagnosed FLAI(E) (*n* = 93)
Median Age (years) (range)	60 (18–75)
Sex	
Male	47 (50.5%)
Female	46 (49.5%)
Secondary disease	
Yes	31 (33.3%)
No	62 (66.7%)
Cytogenetic risk (according to ELN 2022)	
High	31 (33.3%)
Intermediate	55 (59.1%)
Low	6 (6.5%)
Unknown	1 (1.1%)
FLT3 ITD	
Wild type	77 (82.8%)
Mutated	16 (17.2%)
NPM1	
Wild type	63 (67.8%)
Mutated	30 (32.2%)
Median TRM score (range)	2,41 (0,07–50,22)
Performance status	
0–1	27 (29.0%)
2–3	66 (71.0%)
Baseline ANC count	
<500/mmc	32 (34.4%)
≥500/mmc	61 (65.6%)
**Post treatment characteristics**	
Median days at risk (range)	21 (8–59)
Censor reason	
Next cycle	82 (88.2%)
Last contact or 90 days observation	5 (5.4%)
Death	6 (6.4%)
Infectious related death	3 (3.2%)
**Infections events**	
Fever	
Yes	84 (90.3%)
No	9 (9.7%)
FUO	
Yes	35/84 (41.7%)
No	49/84 (58.3%)
Microbiologically documented infections	45 (48.4%)
Bacterial	34 (36.6%)
Gram negative	15 (16.1%)
Gram positive	19 (20.4%)
Virus	1 (1.1%)
Fungal	10 (10.8%)
Radiologically documented infections	23 (24.7%)
Bacterial	11 (11.8%)
Virus	2 (2.2%)
Fungal	10 (10.8%)
Radiologically documented only	4 (4.3%)
Site of infection	
Bloodstream	30 (32.2%)
Upper respiratory tract	3 (3.2%)
Lower respiratory tract	21 (22.6%)
Gastrointestinal	5 (5.4%)
Skin	1 (1.1%)
Central nervous system	0
Genito-urinary	1 (1.1%)
Infectious related ICU admission	1 (1.1%)

*ELN* European Leukemia Net, *TRM* treatment related mortality, *ANC* absolute neutrophil count, *FUO* fever of unknown origin, *ICU* intensive care unit.

Bacterial bloodstream infections were the most frequent and documented in 30/93 (32.3%) patients followed by lower respiratory tract infections observed in 21/93 (22.6%).

Finally, among 6/93 (6.4%) patients who died, 3/93 (3.2%) had an infectious related death: one for a gram-negative septic shock and two for an Aspergillus Pneumonia while on posaconazole prophylaxis oral suspension. The remaining 3/93 (3.2%) patients died for progressive disease. Only one patient (1.1%) was admitted to intensive care unit (ICU).

In our population we did not find any baseline factors statistically associated with increased rates of overall infections, including severe baseline neutropenia. Comparing our ND FLAI(E) to Walti et al. ND CLAG-M, the incidence of fever and infections (microbiologically and/or radiologically documented) was similar, but we observed a higher incidence of bloodstream infections.

We noticed a slightly higher mortality rate in our cohort probably due to the worse performance status of our population. Despite that, we report a lower rate of ICU admission and a similar incidence of infectious related death, notably not related to bacterial infections but to invasive fungal infections (IFI). We also noted a drastic reduction of IFI after the availability of posaconazole oral tablets (2/10), compared to the oral suspension (8/10). Fluoroquinolone prophylaxis still has grade A recommendation [2] but there are increasing warnings about its use in the areas with high fluoroquinolone resistance, like Italy. Indeed, the overuse of antibacterial drugs for prolonged time induces changes in the microbiome composition causing dysbiosis, a condition that promotes further infection complications [3]. In our cohort omitting prophylaxis had probably caused a similar fever rate but a higher incidence of bloodstream infections. However, most of our patients proceeded to next cycle of chemotherapy, indicating that morbidity was not increased.

With the limitations of a retrospective study, our results support the safety of avoiding fluoroquinolone prophylaxis in AML patients treated with FLAI(E), although we think it is mandatory to have a highly efficient protocol for prompt treatment of neutropenic fever in inpatients.

## Data Availability

The datasets generated and analyzed for this study are available from the corresponding author upon reasonable request.
